# Isolated Ocular Manifestations in Chronic Myeloid Leukaemia

**DOI:** 10.7759/cureus.19450

**Published:** 2021-11-10

**Authors:** Anita Maniam, Hanis Zuhaimy, Francesca Martina M Vendargon, Othmaliza Othman

**Affiliations:** 1 Department of Ophthalmology, Universiti Kebangsaan Malaysia Medical Centre, Kuala Lumpur, MYS; 2 Department of Ophthalmology, Hospital Sultanah Aminah, Johor Bahru, MYS

**Keywords:** irradiation, chemotherapy, orbital mass, retinal haemorrhage, chronic myeloid leukaemia (cml)

## Abstract

Background: Chronic myeloid leukaemia (CML) presenting with only ocular manifestations either at the initial stage of diagnosis or at relapse is uncommon. We report two cases of CML presenting with isolated visual symptoms.

Case series: The first case is a 21-year-old healthy gentleman who presented with left eye painless loss of vision for a one-week duration. Visual acuity was 6/60 in the left eye and 6/6 in the right eye. There were scattered retinal haemorrhages in both eyes and a sub-macular bleed over the left eye. The full blood count revealed a high white cell count of 134.6 × 10^9^/L. Peripheral blood smear showed hyper-leucocytosis with absolute eosinophilia and basophilia and the presence of blasts suggestive of CML thus chemotherapy was commenced. The second case is a 28-year-old in haematological, molecular, and cytogenic remission from CML for the past two years, presented with left eye painless vision loss for five days duration. Vision in the left eye was counting fingers. There was a large subretinal mass involving the left optic disc. Magnetic resonance imaging of the brain and orbit showed an elliptical orbital mass at the left globe posteriorly with diffuse thickening of the optic nerve. The patient was diagnosed as CML relapsed to the left optic nerve. He underwent intrathecal chemotherapy and orbital irradiation.

Conclusion: Both these cases are unique since the manifestation of CML was with only ocular features at the time of presentation as per in the first case during the initial diagnosis and in the second case during relapse. This highlights that it is evident that the knowledge of ocular involvement in leukaemia is crucial since the eye is the only organ where leukemic infiltration to nerves and blood vessels can be observed directly. Recognizing fundus changes in leukaemia allows earlier diagnosis and prompt treatment. These case reports highlight the importance of recognizing early fundus changes, which should allow earlier diagnosis and treatment.

## Introduction

Chronic myeloid leukaemia (CML) is a malignant proliferative disorder involving the bone marrow and lymphatic system. It leads to neoplastic white blood cells spreading throughout the bloodstream ultimately affecting organs and tissues. Ocular and orbital involvement in CML has long been recognized. Patients with the ocular manifestation of CML have been reported to have lower five-year survival than those without [1]. In most patients, a diagnosis of CML has been made prior to presentation to an ophthalmologist. It is unusual for ocular manifestations to be the initial presenting sign for the diagnosis of CML as studies have shown that only 5-10% of CML patients present with eye symptoms at initial diagnosis and even rarer to be the initial feature for CML relapse [2]. Here, we report two unique cases of chronic myeloid leukaemia who presented with purely ocular manifestations prior to their diagnosis and relapse, respectively.

## Case presentation

Case 1

**Figure 1:**
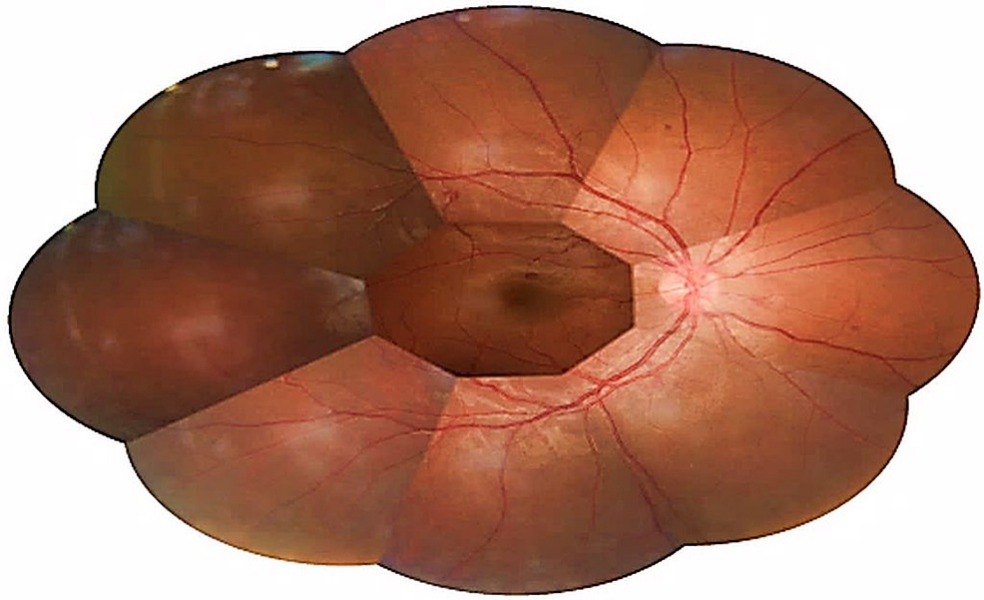
Fundus photo of the right eye showed retinal haemorrhages within the perifoveal area in the first case.

**Figure 2:**
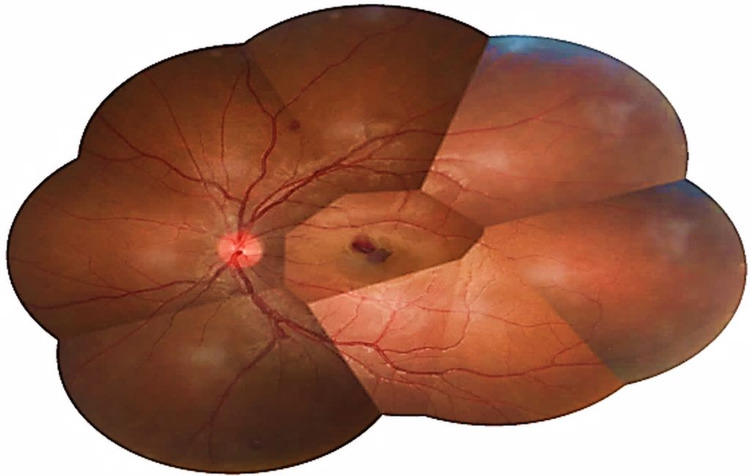
Fundus photo of the left eye showed the presence of retinal haemorrhages within the perifoveal area with a sub-macular bleed, measuring approximately half of the optic disc diameter in the first case.

A 21-years-old Chinese gentleman, with no known medical illness, presented with sudden onset of left eye painless blurring of vision of one-week duration. The patient denied other ocular symptoms such as flashes, floaters, or metamorphopsia. There was no history of ocular trauma. He also denied any constitutional symptoms, autoimmune symptoms, or bleeding tendencies. There was no history of sexual promiscuity or intravenous drug abuse. There was no significant family history of cardiac disease, diabetes, hypertension, or malignancies. He was a non-smoker. On examination, visual acuity was 6/6 and 6/60 in the right and left eye, respectively. Anterior segment assessment in both eyes showed no abnormalities. Fundi examination showed the presence of retinal haemorrhages within the perifoveal area in both eyes (Figures 1 and 2). The left eye had a submacular bleed measuring the approximately one-quarter size of the optic disc (Figure 2). However, there were no Roth’s spots, cotton wool spots, or vasculitis seen in both eyes. Systemic examinations were unremarkable for any lymphadenopathy, bruising, bony tenderness, or hepatosplenomegaly. His vital signs and random blood sugar were normal. An extensive haematological and infectious disease workout was performed. Full blood count showed a high white blood cell count of 134.6 × 10^9^/L (normal value range is 4.00-11.00 × 10^9^/L) which was predominantly neutrophils 121.6 × 10^9^/L (normal value range is 2.00-7.50 × 10^9^/L) about 89.6% and other white cell differentials were lymphocytes 4.3 × 10^9^/L (normal value range is 1.5-4.00 × 10^9^/L) about 3.1%, monocytes 6.9 × 10^9^/L (normal value 0.2-0.8 × 10^9^/L) about 5.1%, eosinophils 1.4 × 10^9^/L (normal value 0.04-0.40 × 10^9^/L) about 1.0% which was high delineating eosinophilia and basophils 1.6 × 10^9^/L (normal value 0.02-0.10 × 10^9^/L) about 1.2% which was high delineating basophilia. His haemoglobin was 11.2 g/dl (normal value range is 11.5-18.6 g/dl) and platelet count was 156 × 10^9^/L (normal value range is 150-400 × 10^9^/L), both were within the normal range. His reticulocyte count was 1.93% (normal value 0.2-2.0%) which was normal. Inflammatory markers; C-reactive protein (CRP) and erythrocyte sedimentation rate (ESR) were slightly raised. CRP measured at 1.2 mg/L (normal value range is less than 1.0 mg/L) while ESR measured at 15 mm/H (normal value range is less than 11 mm/H). Hepatitis B and C serology, venereal disease research laboratory test, and human immunodeficiency virus serology were found to be non-reactive. Rheumatoid factor and antinuclear antibody were negative. Serum lactate dehydrogenase (LDH) was elevated twofold valuing at 412 U/L (normal value range is 135-214 U/L). Other investigations such as fasting serum lipid, renal profile, liver function test, and coagulation profile were all normal. Peripheral blood smear revealed hyperleukocytosis with a bimodal peak of maturation seen, eosinophilia and basophilia. There was the presence of blasts accounting for 1% of total white cells suggestive of CML in the chronic phase. The patient was found to be cytogenetically positive for the Philadelphia (Ph+) chromosome in fluorescence in situ hybridization (FISH) for the BCR-ABL in peripheral blood and bone marrow aspirate. The patient was diagnosed with leukocytosis consistent with CML. He was started on cytoreductive therapy consisting of oral hydroxyurea 1 g twice a day and oral allopurinol 300 mg once a day for two weeks by haematology team. The patient was started with oral Imatinib mesylate 400 mg daily. Upon further follow-up six months later, the vision was 6/6 in the right eye and improved from 6/60 to 6/24 in the left eye with a resolution of the retinal haemorrhages bilaterally. The patient achieved complete haematological and cytogenic remission one year after initiation of Imatinib mesylate therapy.

Case 2

A 28-year-old Chinese gentleman with a known diagnosis of CML and in complete remission for the past two years presented with sudden onset left eye painless loss of vision for five days duration. He initially presented four years ago with a one-month history of prolonged fever and unexplained loss of weight. He underwent extensive hematological workouts and was diagnosed with CML following a high white cell count of 95.3 × 10^9^/L (normal value range is 4.00-11.00 × 10^9^/L) which was predominantly neutrophil 82.1 × 10^9^/L (normal value range is 2.00-7.50 × 10^9^/L) about 86.1%. He had anemia with low haemoglobin of 9.2 g/dl (normal value range is 11.5-18.6 g/dl) and thrombocytosis with a high platelet count of 896 × 10^9^/L (normal value range is 150-400 × 10^9^/L). His peripheral blood showed normocytic normochromic anaemia, marked neutrophilic leucocytosis, thrombocytosis with the presence of blasts cells, and positive bone marrow biopsy with hypercellular marrow suggestive of CML in the chronic phase. He attained haematological, molecular, and cytogenic remission for two years following oral Imatinib mesylate 400 mg daily.

**Figure 3:**
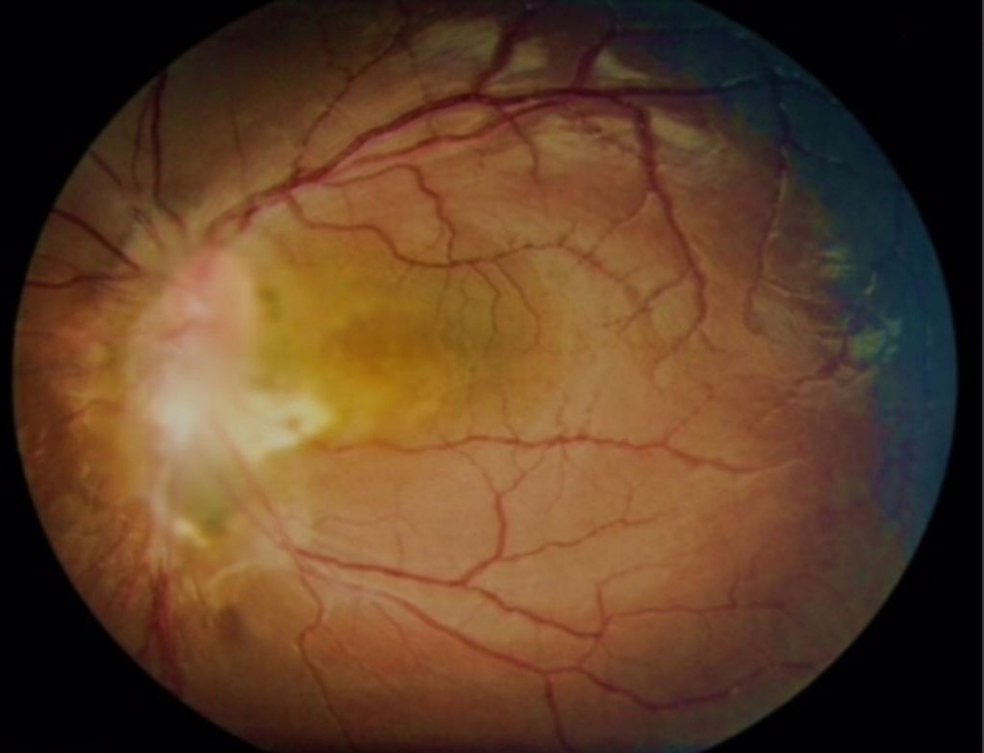
Fundus photo of the left eye showing a large subretinal mass covering the optic disc in the second case.

On examination, his corrected visual acuity was 6/9 in the right eye and counting finger in the left eye. There was a relative afferent pupillary defect in the left eye. As for optic nerve function assessment, there was reduced red saturation and reduced light brightness of about 80% over the left eye. Ishihara test and confrontation of visual field examination over left eye were not done in view of poor vision. Optic nerve function assessment over the right eye was normal. Anterior segments of both eyes were normal. Fundus examination of the left eye revealed a large pale subretinal mass overlying the optic disc measuring 7.2 mm (vertical) × 6.6 mm (horizontal). There was no vitritis, vitreous, or retinal haemorrhages and the retina was flat (Figure 3). Fundus examination of the right eye was unremarkable with the normal optic disc, macula, and retina. Systemic examination was unremarkable for lymphadenopathy, bruising, bony tenderness, or hepatosplenomegaly. Neurological examination was normal.

**Figure 4:**
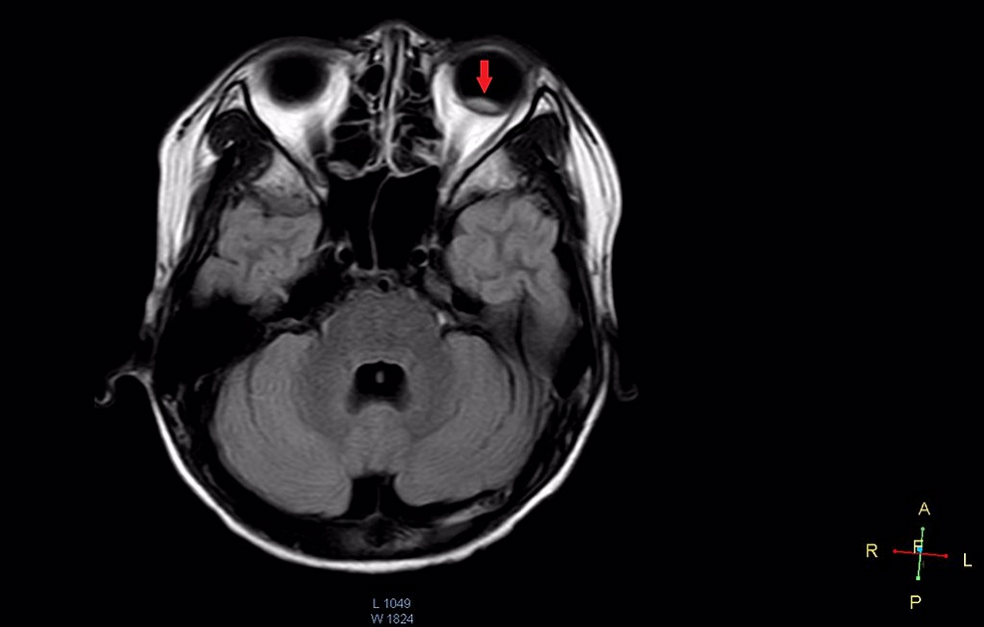
Magnetic resonance imaging of brain and orbit showing an elliptical lesion at the posterior aspect of the left globe with diffuse thickening of the left optic nerve in the second case.

Magnetic resonance imaging (MRI) of the brain and orbit showed an elliptical lesion at the posterior aspect of the left globe measuring 0.4 cm (antero-posterior, AP) × 0.8 cm (transverse, TV) × 0.7 cm (craniocaudal, CC) with diffuse thickening of the left optic nerve (Figure 4). The lesion was hypointense in the T1-weighted image and hyperintense in fluid-attenuated inversion recovery (FLAIR) with enhancement in the post-contrast study. The right globe was unremarkable. There were no focal enhancing lesions noted in the brain parenchyma. A lumbar puncture was performed by haematology team. Cerebrospinal fluid (CSF) analysis revealed normal biochemistry parameters and cytology. There were no blast cells in CSF seen. Bone marrow aspirate was negative for the Philadelphia chromosome. A diagnosis of CML relapse with leukemic infiltration to the left optic nerve and orbit was made. He was immediately given weekly intrathecal Methotrexate, Cytarabine, and Hydrocortisone chemotherapy regimen. The patient received oral Dasatinib 150 mg daily. Radiation therapy was initiated over 10 days encompassing left orbit with 2000 centiGray (cGy) and an additional boost of 900 cGy to his left posterior retina and optic nerves. After six months of initiation of treatment, the patient attained complete cytogenetic response without signs of systemic or central nervous system relapses. However, the vision over the left eye remained impaired and the optic disc was pale suggesting optic disc atrophy with inferotemporal perivascular sheathing. Repeated MRI of brain and orbit after six months of the second initiation of anti-leukemic therapy showed left optic nerve atrophy with a resolution of posterior globe mass, the right globe was normal, and there were no focal enhancing lesions noted in the brain parenchyma.

## Discussion

Leukaemias are classified by the formation of abnormal leukocytes and by how rapidly these cells leave the bone marrow and invade the bloodstream. The four main types of leukaemia are acute myeloid leukaemia, CML, acute lymphoblastic leukaemia, and chronic lymphocytic leukaemia.

Ocular involvement in leukaemia can precede the diagnosis of leukaemia or can occur during the disease course itself. Ocular-related symptoms may sometimes serve as the initial mode of presentation of leukaemia in 3.6% of patients and in 39% of patients it occurred as a secondary complication [3]. Ocular involvement is infrequently seen in chronic forms of leukaemia than in acute forms [4]. Ocular complications of leukaemia are known to be due to direct infiltration of the orbit and other ocular tissues such as iris, choroid, optic nerve, or as a result of vascular abnormalities affecting the retina exhibiting as leukemic retinopathy. Prolonged leucocytosis in CML causes an increased number of circulating platelets leading to increased whole blood viscosity which paves way for reduced blood flow and vascular stagnation causing peripheral capillary dropout and microaneurysm formation which eventually causes proliferative retinopathy [5]. Leukaemic retinopathy is characterized by the presence of multiple preretinal, intraretinal, or subretinal haemorrhages that are most notably present in the posterior pole. Other clinical signs include cotton wool spots, hard exudates, Roth’s spots, perivascular sheathing, retinal venous tortuosity, and neovascularization. Our first patient had retinal hemorrhages confined to the posterior pole in fundi examination which are features of leukemic retinopathy. Retinal involvement in fact is a rare form of presentation of CML and few cases have been reported so far [5]. Furthermore, leukaemic infiltration causes fulminant disease and early mortality with clinical presentations such as large greyish white nodules of varying sizes in the retina, grey-white streaks along vessels caused by local perivascular infiltrates, subretinal infiltrates which is also referred to as subretinal hypopyon, optic nerve infiltration, choroidal infiltrates causing serous retinal detachments, retina pigment epithelium (RPE) detachments, discrete choroidal masses, and vitreous infiltrates [6].

Clinical presentation of optic nerve infiltration varies in CML. Optic disc swelling with or without evidence of optic nerve dysfunction and normal appearance of optic disc associated with evidence of dysfunction have been reported [7]. Optic disc swelling is the most commonly reported sign of optic nerve infiltration in CML. It occurs as a result of direct infiltration of the nerve by leukemic cells, increased intraocular pressure, or swelling due to retro laminar leukemic invasion. Extra-medullary relapse presenting as isolated blindness from optic nerve infiltration is extremely rare, occurring more frequently in children with acute lymphoblastic leukemia than in adults with CML [1]. However, there are a few reported cases in the past of adult patients with relapse CML presenting with isolated ocular signs. Gulati et al. reported a case of a 66-year-old man with relapsed CML to the optic nerve and choroid which was similar to our second case [8]. Our patient from the second case had a large subretinal mass covering the left optic disc associated with left optic nerve dysfunction. His MRI of the brain and orbit concluded that there was a left orbital mass likely to represent leukemic infiltration with extension into the left optic nerve.

Through investigations to rule out neurological or systemic leukemic relapse should be undertaken promptly. Our first case had undergone detailed investigations and the diagnosis was made early. He received chemotherapy early in his disease before other systemic manifestations of CML were evident. He responded well and is remission-free till now. Our second case had undergone a lumbar puncture and CSF analysis to look for the presence of blast cells. Surprisingly, the CSF fluid showed normal cytological findings and was negative for blast cells. The bone marrow aspirate was negative for the Philadelphia chromosome. The patient was immediately given intracranial radiation for treating the optic nerve infiltration of which he responded. The second case-patient received also received intrathecal chemotherapy for prophylaxis of CNS involvement considering the close proximity of CSF and optic nerve. The region of the body where leukemic cells are relatively protected from the cytolytic effects of systemic chemotherapy is recognised as a pharmacologic sanctuary. The optic nerve is considered as a pharmacologic sanctuary from systemic chemotherapy hence isolated invasion of the optic nerve may occur as the optic nerve is poorly penetrated by systemic chemotherapy [9]. It is postulated that there could be a barrier between the central nervous system and the optic nerve lesions in patients with leukemic infiltration of the optic nerve. The barrier disrupts the smooth flow of CSF, hence the chemotherapeutic drugs cannot reach the affected areas of the optic nerve [10]. Focal irradiation is significantly effective as it reduces the leukaemic cell in the optic canal and this allows the chemotherapeutic drugs in the CSF to attack the neoplastic cells.

It has been published that the five-year survival of patients with ophthalmic manifestation or infiltration of CML was 21.4% compared to 45.7% of those patients without [1]. Traditionally, patients with ocular leukemia are treated with systemic chemotherapy and biological treatments [11]. Imatinib is a selective Bcr-Abl tyrosine kinase inhibitor (TKI) which has been the standard therapy and first-line treatment for CML due to its notable activity and mild toxicity. The International Randomized Study of Interferon and STI571 (IRIS) reported that the estimated 10-year overall survival of Imatinib-treated CML patients was 83.3% [12]. Imatinib can attain major cytogenetic response in more than 60% of CML patients and haematological response in 95% of CML patients [13]. It has been reported previously that leukaemic retinopathy was successfully treated with Imatinib [14]. In a review encompassing 40 published cases that described ocular and ophthalmic manifestations in patients with CML, in 17 cases Imatinib was used as the TKI, two cases used Bosutinib, and another two cases used Nilotinib [15]. Hence, Yassin et al. reported that Imatinib is the most prescribed TKI in CML with ocular and ophthalmic manifestation probably because it was the first available TKI and has been recommended as first-line CML treatment; however, the outcome of the TKI usage in CML with ocular and ophthalmic manifestation was not analysed further due to inadequate data especially regarding the risk score and the prognostic factor in the published cases [15]. Our patient from the first case was started on Imatinib and he attained complete haematological and cytogenic remission one year with a resolution of his CML retinopathy. Although Imatinib is the first-line treatment for CML, several studies have shown that the penetration of the drug into the CNS is poor [16]. Dasatinib has more potency than Imatinib and passes through the blood-brain barrier to reach the CSF [17]. Satake et al. report a leukaemic case with optic nerve infiltration being successfully treated with Dasatinib [18]. Similarly, the patient in the second case was started with oral Dasatinib and he responded well. CML patients with optic nerve infiltration as seen in our second case require intrathecal chemotherapy and orbital radiation. Leukemic cells are radiosensitive. Although there is some risk associated with radiation therapy near the optic nerve, a course of 2000 cGy over a one to two-week period is a typical regimen and may result in dramatic resolution of infiltration [19]. Intrathecal chemotherapy alone however may yield limited treatment efficacy. Recently, a retrospective cohort study by Vishnevskia et al. stated that intravitreal methotrexate injections may be an effective mode of treatment for eyes with intraocular leukaemic tumour cell infiltration whereby in their study 11 treated eyes showed improvement in the inflammatory reaction and tumour cell infiltration and this could be a promising therapeutic approach in future [20].

## Conclusions

In conclusion, these two cases highlight the importance of a detailed eye assessment in patients with CML who do not present with the typical systemic signs and symptoms of CML. It is evident that the knowledge of ocular involvement in leukaemia is imperative since the eye is the sole site where the leukemic infiltration can be observed directly. If leukaemic infiltration is evident, treatment should be promptly initiated potentially involving cytoreduction with chemotherapy, initiation of TKI, and orbital radiation with the goal of preserving vision and the globe.

## References

[1] Ohkoshi K, Tsiaras WG (1992). Prognostic importance of ophthalmic manifestations in childhood leukaemia. Br J Ophthalmol.

[2] Guyer DR, Schachat AP, Vitale S (1989). Leukaemic retinopathy. Relationship between fundus lesions and haematologic parameters at diagnosis. Ophthalmology.

[3] Chaudhuri T, Roy S, Roy P (2013). Ischaemic optic neuropathy induced sudden blindness as an initial presentation of acute lymphoblastic leukemia. Indian J Med Paediatr Oncol.

[4] Koshy J, John MJ, Thomas S, Kaur G, Batra N, Xavier WJ (2015). Ophthalmic manifestations of acute and chronic leukemias presenting to a tertiary care center in India. Indian J Ophthalmol.

[5] Huynh TH, Johnson MW, Hackel RE (2007). Bilateral proliferative retinopathy in chronic myelogenous leukemia. Retina.

[6] Reddy SC, Jackson N (2004). Retinopathy in acute leukaemia at initial diagnosis: correlation of fundus lesions and haematological parameters. Acta Ophthalmol Scand.

[7] Miller NR, Walsh FB, Hoyt WF (2005). Walsh and Hoyt's Clinical Neuro-Ophthalmology.

[8] Gulati R, Alkhatib Y, Donthireddy V, Felicella MM, Menon MP, Inamdar KV (2015). Isolated ocular manifestation of relapsed chronic myelogenous leukemia presenting as myeloid blast crisis in a patient on imatinib therapy: a case report and review of the literature. Case Rep Pathol.

[9] da Costa DR, Fernandes RD, Susanna FN, da Silva Neto ED, Monteiro ML (2021). Complete reversal of bilateral optic nerve infiltration from lymphoblastic leukemia using chemotherapy without adjuvant radiotherapy. BMC Ophthalmol.

[10] Lin YC, Wang AG, Yen MY, Hsu WM (2004). Leukaemic infiltration of the optic nerve as the initial manifestation of leukaemic relapse. Eye (Lond).

[11] Wolff-Kormann PG, Hasenfratz BC, Heinrich B, Kormann B (1991). Successful treatment with hydroxyurea of ocular involvement in chronic myelomonocytic leukemia. N Engl J Med.

[12] Hochhaus A, Larson RA, Guilhot F (2017). Long-term outcomes of imatinib treatment for chronic myeloid leukemia. N Engl J Med.

[13] Altintas A, Cil T, Kilinc I, Kaplan MA, Ayyildiz O (2007). Central nervous system blastic crisis in chronic myeloid leukemia on imatinib mesylate therapy: a case report. J Neurooncol.

[14] John BC, Daniel JD, Donald JD (2003). Resolution of leukemic retinopathy following treatment with imatinib mesylate for chronic myelogenous leukemia. American Journal of Ophthalmology.

[15] Yassin MA, Ata F, Mohamed SF (2021). Ophthalmologic manifestations as the initial presentation of chronic myeloid leukemia: a review [PREPRINT]. Surv Ophthalmol.

[16] Neville K, Parise RA, Thompson P (2004). Plasma and cerebrospinal fluid pharmacokinetics of imatinib after administration to nonhuman primates. Clin Cancer Res.

[17] Lombardo LJ, Lee FY, Chen P (2004). Discovery of N-(2-chloro-6-methyl- phenyl)-2-(6-(4-(2-hydroxyethyl)- piperazin-1-yl)-2-methylpyrimidin-4- ylamino)thiazole-5-carboxamide (BMS-354825), a dual Src/Abl kinase inhibitor with potent antitumor activity in preclinical assays. J Med Chem.

[18] Satake A, Okada M, Asada T (2010). Dasatinib is effective against optic nerve infiltration of Philadelphia chromosome-positive acute lymphoblastic leukemia. Leuk Lymphoma.

[19] Nagpal MP, Mehrotra NS, Mehta RC, Shukla CK (2016). Leukemic optic nerve infiltration in a patient with acute lymphoblastic leukemia. Retin Cases Brief Rep.

[20] Vishnevskia-Dai V, Sella King S, Lekach R, Fabian ID, Zloto O (2020). Ocular manifestations of leukemia and results of treatment with intravitreal methotrexate. Sci Rep.

